# Long-acting reversible contraception with etonogestrel implants in female macaques (*Macaca mulatta* and *Macaca fascicularis*)

**DOI:** 10.3389/fvets.2023.1319862

**Published:** 2024-01-08

**Authors:** Annemiek Maaskant, Kimberly K. Scarsi, Lisette Meijer, Sandra Roubos, Annet L. Louwerse, Edmond J. Remarque, Jan A. M. Langermans, Marieke A. Stammes, Jaco Bakker

**Affiliations:** ^1^Biomedical Primate Research Centre, Rijswijk, Netherlands; ^2^Department Population Health Sciences, Animals in Science and Society, Faculty of Veterinary Medicine, Utrecht University, Utrecht, Netherlands; ^3^Department of Pharmacy Practice and Science, College of Pharmacy, University of Nebraska Medical Center, Omaha, NE, United States

**Keywords:** contraception, etonogestrel, Implanon, fertility, long-acting contraception, macaques, reversibility, non-human primates

## Abstract

**Introduction:**

Contraception is often required for management and population control purposes in group-housed and free-roaming non-human primates. Long-acting reversible contraceptives, including subdermal progestin-releasing implants, are preferred as they eliminate challenges associated with frequent administration. Etonogestrel (ENG)-releasing subdermal implants are reversible and long-acting for a minimum of 3 years, and are commercially available for human use as Implanon^®^ or Nexplanon^®^.

**Methods:**

A retrospective analysis was performed detailing the contraceptive effectiveness and reversibility of subdermal placement of one-fourth or one-third of an ENG implant (68 mg/implant) in 129 female rhesus macaques (*Macaca mulatta*) and 67 cynomolgus macaques (*Macaca fascicularis*) at the Biomedical Primate Research Centre (Rijswijk, Netherlands). Furthermore, single cross-sectional ENG serum concentrations were measured for 16 rhesus and 10 cynomolgus macaques, and hemoglobin and blood chemistry pre-ENG and at timepoints >0.5, >1.5, and > 2.5 years post-ENG insertion were evaluated for 24 rhesus macaques. Finally, data were obtained using trans-abdominal ultrasound regarding the influence of ENG on uterine volume and endometrial thickness in 14 rhesus and 11 cynomolgus macaques.

**Results:**

As a contraceptive ENG was in 99.80% (CI 93.50–99.99) and 99.95% (CI 99.95–100) effective in rhesus and cynomolgus macaques, respectively. Prolonged ENG durations of implant use in 14 rhesus macaques (range 3.1–5.0 years) and eight cynomolgus macaques (range 3.2–4.0 years) resulted in no unintended pregnancies. A total of 17 female macaques were allowed to breed after ENG removal, and among them, 14 female macaques (82%) had an uneventful delivery. Serum ENG concentrations with a median ENG duration of 1.2 years (range 0.1–6.0 years) and 1.9 years (range 0.6–4.7 years) resulted in median concentrations of 112 pg./mL (range 0–305 pg./mL) and 310 pg./mL (range 183–382 pg./mL) for rhesus and cynomolgus macaques, respectively. ENG had no clinical effect on hemoglobin and blood chemistry parameters nor on the thickness of the endometrial lining or uterus volume.

**Conclusion:**

This study indicates that both one-fourth and one-third of the ENG implants are effective, long-acting, reversible, and safe contraceptive to use in macaques.

## 1 Introduction

Contraception is often required for population control of free-roaming non-human primates and management purposes in captive group-housed non-human primates (NHPs). Frequent individual oral treatment is challenging and unreliable in both free-roaming and captive-housed macaques whereas surgical intervention is reliable but requires an invasive intervention and is in principle irreversible (1, 2). Therefore, minimal invasive long-acting reversible contraceptives are preferred as they eliminate problems associated with daily, weekly, or monthly administration.

In humans, subdermal progestin-only implants releasing etonogestrel (ENG) (i.e., Implanon^®^, and Nexplanon^®^) are described to provide long-acting reversible contraception (3–7). In humans, ENG prevents pregnancy by suppressing the secretion of luteinizing hormones, thickening the cervical mucus, and causing the endometrial lining to become thin and atrophic, i.e., preventing ovulation and decreasing penetration of spermatozoa into the uterus and preventing uterine attachment of the conceptus. ENG concentration of 90 pg./mL has been described as a threshold level to prevent ovulation (8). Implanon^®^ is known to be more than 99% effective for at least 3 years; however, prolonged use of up to 5 years has been described as well (9–11).

In addition, other studies show that the effectiveness of ENG seems independent of body weight. The overall contraceptive effectivity and ENG concentrations in overweight and obese women were similar compared to women with a lower body mass index (BMI) when inserted with one implant (12–14). However, the product labeling suggests that ENG should not be used in woman >130% of their ideal body weight. In addition to ENG effectiveness, it is well established from a variety of human studies that ENG has minor effects on hematological, biochemical, and hormonal parameters. As both rhesus and cynomolgus macaques have proven to be good models for preclinical testing of (human) female reproductive medicine, the authors assume that the effects of ENG in macaques will be in line with the presented human literature (15).

Although various studies have been carried out on the use of ENG in humans, literature concerning the use of ENG in NHPs is limited (16–18). In addition, these studies do not present details regarding optimal dose, serum levels, contraceptive effectivity, reversibility, or effect on the reproductive organs of ENG in macaques. However, ENG has been successfully used for years as a contraceptive in macaques at the Biomedical Primate Research Centre (BPRC, Rijswijk, Netherlands).

The objective of this study was to fill this gap of knowledge and present recommendations to provide optimum contraception in macaques. Therefore, a retrospective data analysis was performed detailing the contraceptive effectivity and reversibility of one-fourth and one-third part of subcutaneous inserted ENG implants in female captive-housed rhesus and cynomolgus macaques. Serum ENG concentrations in both species were assessed, routine hematology and chemistry parameters pre- and post-ENG implant were evaluated, and a trans-abdominal ultrasound was performed to obtain data regarding the influence of ENG on uterine volume and thickness of the endometrial lining.

## 2 Materials and methods

### 2.1 Animals, housing, and husbandry

Female rhesus macaques (*Macaca mulatta, n* = 140) and cynomolgus macaques (*Macaca fascicularis*, *n* = 70) receiving ENG were analyzed retrospectively. The BPRC houses an outbred breeding colony of both species in which the animals are housed in multigenerational family groups in enriched enclosures, consisting of freely accessible indoor and outdoor compartments. The indoor and outdoor enclosures had a floor surface of 72 m^2^ and 208 m^2^, respectively. The floor of the indoor enclosure was provided with wood fiber bedding, while the outside enclosure consisted of sand bedding. Environmental enrichment consisted of several climbing structures, beams, fire hoses, car tires, sitting platforms, and a swimming pool to facilitate natural behavior. Indoor enclosure temperature was 18°C and 21°C for rhesus and cynomolgus macaques, respectively, with a 12-h light–dark cycle.

The animals were fed with commercial monkey pellets (Ssniff, Soest, Germany) supplemented with limited amounts of fruit, vegetables, or grain mixtures. Water was available *ad libitum* provided by automatic water dispensers.

Qualified animal caretakers observed all animals at least twice daily for injuries and illnesses. In addition, signs of any abnormalities specific to this study, including abnormal sexual behavior and abnormal vaginal excretions, were reported to the veterinarians. Health and medical records were kept for each animal.

As part of the BPRC health surveillance, all animals of the colony underwent annual health evaluations. These annual examinations were performed and distributed over the year, with a planned interval of 12 months for each animal. For these evaluations, the animals were fasted overnight while water remained available. All evaluations were performed in the morning. Until 2019, general anesthesia was induced and maintained solely with an intramuscular injection of 10 mg/kg ketamine (ketamine 10%; Alfasan Nederland BV, Woerden, Netherlands). From 2020 onward, medetomidine (0.05 mg/kg intramuscular, Sedastart; AST Farma BV, Oudewater, Netherlands) was added to the ketamine injection. Body weight was recorded, and blood samples were collected for standard hematology (HER) and chemistry (CER) analyses. In addition, a thorough physical examination and a pregnancy test using abdominal palpation or ultrasound were performed by a veterinarian. Furthermore, some animals received ENG for colony management or veterinary reasons, such as dysmenorrhea or presumptive endometriosis. None of the animals received ENG solely for the purpose of this study. After finishing the entire examination, the animals were returned to their enclosure, and intramuscular atipamezole (0.25 mg/kg, Sedastop; AST Farma BV, Oudewater, Netherlands) was administered.

All animals were housed in accordance with Dutch law and international ethical and scientific standards and guidelines (EU Directive 63/2010). All procedures and husbandry were compliant with the above standards and legislations. No interventions other than those required for the regular veterinary examination were performed on these animals. Therefore, in accordance with the law on animal experiments and the EU directive, no approval from the competent authorities was required. Nevertheless, additional approval was obtained from the institutional animal welfare body (IvD 023A). The BPRC is accredited by the Association for Assessment and Accreditation of Laboratory Animal Care (AAALAC) International.

### 2.2 Implant

The contraceptive effectiveness, dose, reversibility, and effect on blood parameters and the uterus in animals receiving ENG implants [available in Europe as Implanon^®^; 68 mg etonogestrel; N.V. Organon International, Oss, Netherlands], were analyzed.

**Figure 1:**
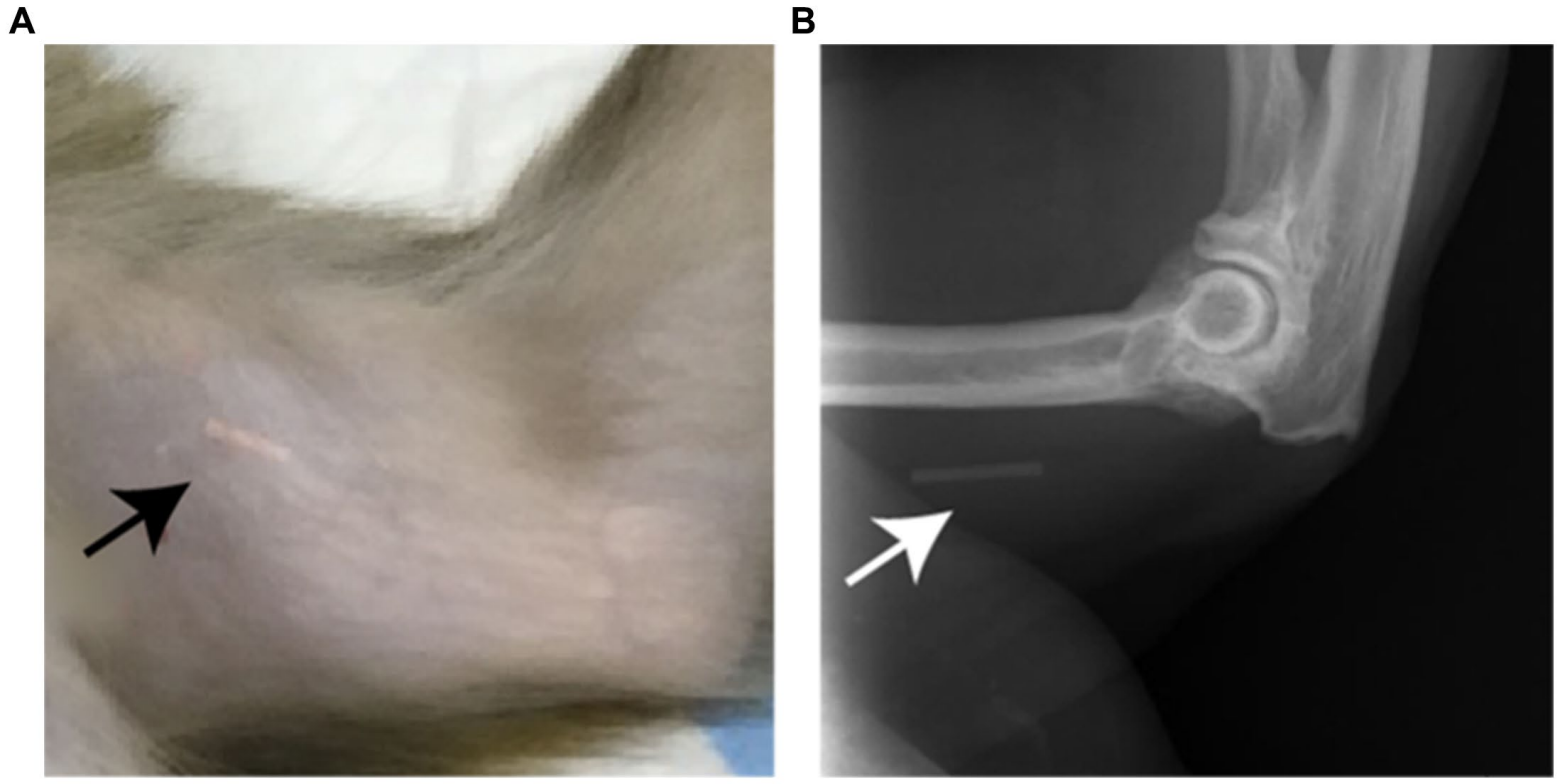
**(A)** Photograph of the subcutaneous located implant, indicated by the arrow, which is visible through the unshaved skin on the medial side of the upper arm of a rhesus macaque. **(B)** on a X-ray image the implant (arrow) is also visible as the implant contains barium sulfate.

The used dose was independent of the weight of the animals; however, an entire implant is not necessary for NHPs. Therefore, the 4-cm-long implant was divided into three or four parts with a sterile scalpel blade. The disposable applicator was manually loaded with the implant part. Subsequently, the insertion site was disinfected (70% alcohol) but not shaved. The ENG part was inserted subcutaneously with the applicator, approximately 8–10 cm (3–4 inches) above the medial epicondyle of the humerus just under the skin, avoiding the sulcus between the biceps and triceps muscles and the large blood vessels and nerves that lie there in the neurovascular bundle deeper in the subcutaneous tissues (Figure 1). The insertion site did not require sutures to be closed. The remaining parts of the implant were stored for a maximum of 6 months in a refrigerator (3–5^o^C) for use in other animals.

Based on the manufacturer’s description, the implant was intended to be removed and replaced, if further contraception was required, after 3 years.

### 2.3 Blood sampling and analysis

The collection site was shaved and sterilized with 70% alcohol. Subsequently, 1 mL EDTA blood for HER analyses (Greiner Bio-One GmbH, Kremsmünster, Austria) and 5 mL clotted blood for CER analyses (Greiner Bio-One GmbH, Kremsmünster, Austria) were collected from the femoral vein using a 20-gauge Vacuette^®^ needle and hub. HER samples were mixed by inversion, whereas CER samples were collected in tubes without anticoagulants and allowed to clot at room temperature for at least 1 h. Chemistry samples were centrifuged at 3,000 rotations per minute (rpm) for 10 min. Hematology was determined using a Sysmex XT2000iV (Sysmex BV, Etten-Leur, Netherlands). Clinical chemistry was determined using a Cobas Integra 400 plus analyzer (F. Hoffmann-La Roche Ltd., Switzerland).

For the retrospective HER and CER evaluation, only animals were included with available non-pregnant baseline HER and CER analyses prior to ENG and at least one in-treatment HER and CER analysis with a minimum of 0.5 years post-ENG insertion. The timepoints were set in ranges of 0.5–1.5 years (>0.5), 1.5–2.5 years (>1.5), and 2.5–3.5 (2.5) years in-treatment. Female animals with presumptive endometriosis, diarrhea, or suspected (pre)diabetes diagnosed during the physical examination were excluded from this analysis.

A volume of 2 mL of serum was collected in cryo vials (Cryo.S PP, Greiner Bio-One GmbH, Kremsmünster, Austria) and stored at −80°C until sample shipment. ENG concentrations were quantified by a validated liquid chromatography–tandem mass spectrometry (LC–MS/MS) method per US Food and Drug Administration criteria.^1^ The range of quantitation was 0.025–30 ng/mL, and the coefficient of variation was less than 15%. For analysis of serum ENG concentration, a single serum sample was obtained from 16 rhesus and 10 cynomolgus macaques with an ENG implant in place during the health evaluations performed between February and November 2021, irrespective of the duration of implant use. Serum from two female control animals without ENG was included as a negative control.

### 2.4 Ultrasound of the uterus

Trans-abdominal ultrasounds of the uterus of non-pregnant female animals with and without ENG were obtained by two imaging scientists during the health evaluations performed between February and November 2021. These scientists were blinded regarding the use of ENG or not.

Non-pregnant female animals, aged ≥3 years, were placed in a supine position, and their caudal abdomen was shaved prior to the application of ultrasound gel. Subsequently, a longitudinal and cross-sectional scan of the entire uterus was obtained with an ACUSON Juniper ultrasound system and micro-convex 11 M3 3.5–11.0 MHz transducer (Siemens Healthcare GmbH, Erlangen, Germany). Based on these scans, the largest cross section was determined to measure the length, width, and height of the uterus to be able to calculate the volume of the uterus. In addition, the thickness of the endometrium was measured (19).

A total of 201 rhesus and 88 cynomolgus macaques were screened with this procedure. Of these animals, 14 rhesus and 11 cynomolgus macaques had an ENG implant in place. Several animals were scanned and measured twice during the same procedure to check and compensate for intra-observer variation. To prevent inaccurate statistical assumptions due to the inequality of the group sizes, the in-treatment animals were group-matched with controls. The parity, age, and season in which the ultrasound was obtained for the rhesus macaques (e.g., breeding season or non-breeding season) were group-matched. If there were multiple similar matches, one individual was randomly selected.

### 2.5 Inclusion criteria

Records from the electronic database were collected from 15 October 2005 to 17 August 2021. Animals were included if all data were available regarding age, date of parturitions after ENG insertion, dose, and date of insertion and removal.

The minimum in-treatment period of ENG for inclusion was 48 days. In the case of gestation, the embryo is then clearly visible with ultrasound, and the enlarged uterus is palpable in the abdomen (20–23). This is an important criterion to eliminate the probability of a false negative pregnancy test, as performed during the annual health examinations.

The length of the ovarian cycle and gestation was set at 28 and 165 days, respectively (15, 22–28). The duration of ENG treatment was defined as the number of days elapsed between implant insertion and its removal or in a few cases euthanasia because of health and welfare considerations.

Criteria for enrollment in the effectiveness calculation section included being housed with a fertile male (age ≥ 3 years) and a clinically healthy reproductive tract, e.g., female animals with impairment of the reproductive tract, such as presumptive endometriosis, were excluded from this analysis. The duration of ENG was based on the manufacturer’s advice of a maximum of 1,095 days (similar to 3 years), and for additional effectiveness calculations of the prolonged in-treatment period, animals were included from 1,096 days and up without renewal of the implant. The extent of exposure to the implant was expressed in both years and total number of in-treatment days. Contraceptive effectiveness was calculated by the number of unintended pregnancies divided by the potential number of ovarian cycles of all implanted animals (6, 29).

In contrast to cynomolgus macaques that breed year-round, rhesus macaques have a breeding season. To prevent overestimation of ENG effectiveness in rhesus macaques, the exposure to male animals was only evaluated during the breeding season, which lasts from October to March at the BPRC (30).

Restoration of reproductive potential following removal of the implant was calculated as well. For this reversibility evaluation of ENG, only those female animals were selected, which were in the presence of a fertile male after ENG removal. In three rhesus macaques, a new male was introduced approximately 6 years after ENG insertion, and in these subjects, the implant was not removed after 3 years.

### 2.6 Data analysis

Statistical tests were performed with R studio v4.1.3 and GraphPad prism v8.4.2. All data were tested non-parametrically. Wilcoxon’s signed-rank test was used to test paired samples of hemoglobin and biochemical blood parameters. To compare differences between independent samples in ENG serum concentrations and ultrasound data, the Mann–Whitney U-tests were used. Possible associations between ENG serum concentrations, ENG duration, and body weight were evaluated with Spearman’s rank correlations. Fisher’s exact test was used to calculate differences in effectiveness between doses, e.g., one-fourth and one-third ENG part. *p*-values < 0.05 were considered statistically significant, and 95% confidence intervals are reported as CI (lower limit-upper limit).

## 3 Results

### 3.1 In-treatment ENG data

The inclusion criteria were met by 129 female rhesus and 67 cynomolgus macaques (Table 1).

**Table 1:** Summary of retrieved database records of the female rhesus and cynomolgus macaques meeting the inclusion criteria for etonogestrel (ENG) evaluation.

	Rhesus macaques	Cynomolgus macaques
Number animals	129	67
Mean age (years) 1st ENG (min-max)	11.0 (3–25)	11.5 (2–23)
Mean parity (min-max)	4 (0–13)	2 (0–7)
Total number of implants	137	108
Range number implants/female	1–3	1–4
Dose		
One-fourth part	46	62
One-third part	91	46
Presumptive endometriosis and other suspected pathology	4	9

Overall, ENG was inserted in 95 multiparous and 34 nulliparous rhesus and in 46 multiparous and 10 nulliparous cynomolgus macaques. The parity was unknown in 11 cynomolgus macaques. The mean duration of ENG implant use was 908 days (2.5 years) and 1,217 days (3.3 years) for rhesus and cynomolgus macaques, respectively, regardless of whether the expiration duration of ENG had exceeded.

### 3.2 Three-year effectiveness

Table 2 shows the data used for the effectiveness calculation. Three-year effectiveness was calculated with an ovarian cycle of 28 days, and only female animals with a treatment duration of <1,096 days were included. As the cutoff point, the last day with a male was used as the end point for the duration.

**Table 2:** Data used to calculate <3-year ENG effectiveness of rhesus and cynomolgus macaques in the presence of a fertile male.

	Rhesus macaques	Cynomolgus macaques
Number of animals	109	52
Total number of ENG	116	87
Total number of cycles	1,460	2,360
Number of cycles in male presence	1,019	2,148
Number of in-treatment pregnancies	2	1
Cycles after pregnancy correction	1,007	2,142
Success rate % (CI)	99.80 (93.5–99.99)	99.95 (99.9–100)

In rhesus macaques, a total of 1,460 in-treatment cycles during the breeding season were observed, and 1,019 cycles were covered by sexually mature males. Two in-treatment rhesus females had an unintended pregnancy, which resulted in uneventful deliveries. The female animals conceived approximately on in-treatment days 16 and 1,026 (2.8 years), demonstrating a effectiveness of 99.80% (CI 93.5–99.99).

In cynomolgus macaques, 2,142 cycles for possible conception in the presence of a fertile male were observed. Only one in-treatment conception occurred approximately 314 days after ENG insertion, demonstrating a effectiveness of 99.9% (CI 99.9–100).

### 3.3 Prolonged 3–5-year effectiveness

Fourteen rhesus females, three animals with one-fourth and 11 animals with one-third ENG, had a prolonged in-treatment period exceeding 1,096 days in the presence of a male, resulting in ENG durations ranging between 1,134 and 1,805 days, with a median period of 1,250 days (range 3.1–5.0, median 3.4 years). In addition, eight cynomolgus macaques, six animals with one-fourth and two with one-third ENG, had a prolonged in-treatment period of 1,154–1,441 days, with a median period of 1,228 days (range 3.2–4.0, median 3.4 years) in the presence of a male. No unintended pregnancies were observed in either species.

### 3.4 Reversibility

In 42 rhesus macaques, the implant was removed, yet none of these animals were allowed to return to breeding status. In 22 cynomolgus macaques, the implant was removed, and 14 animals were allowed to breed. In three rhesus macaques, the implant was not removed; however, the implant was 5.9 years *in situ* when a new breeding male was introduced. This resulted in parturitions at 6.3, 6.4, and 6.5 years post-ENG insertion. These three dams gave birth to viable neonates at 177, 190, and 229 days after the start of the introduction of the new alpha male.

All 14 cynomolgus macaques, which were allowed to breed, became pregnant. For nine females, there was already a male present in their social group at the time of ENG removal, resulting in parturitions ranging between 184 and 393 days, with a median period of 243 days (range 0.5–1.1, median 0.67 years) after removal. The other five females had a parturition between 172 and 330 days (median 349 days) after the presence/introduction of a male. Eleven female animals produced viable neonates, one female underwent a cesarean section because of an incomplete cervical dilation with a dead emphysematous fetus, and two neonates were found dead in the morning after parturition. Both species together resulted in 14 of 17 (82%) uneventful deliveries compared to a yearly estimated 90% of uneventful deliveries in the whole breeding colony (data not shown).

### 3.5 Dose

In 91 rhesus and 46 cynomolgus macaques, the dose was one-third ENG implant while 46 rhesus and 62 cynomolgus macaques received one-fourth ENG implant. No difference was observed between one-fourth or one-third ENG implant and contraceptive efficacy in rhesus and cynomolgus macaques (*p* = 0.99 and *p* = 0.51), respectively (two-tailed Fisher’s exact test).

### 3.6 ENG serum concentration

**Figure 2:**
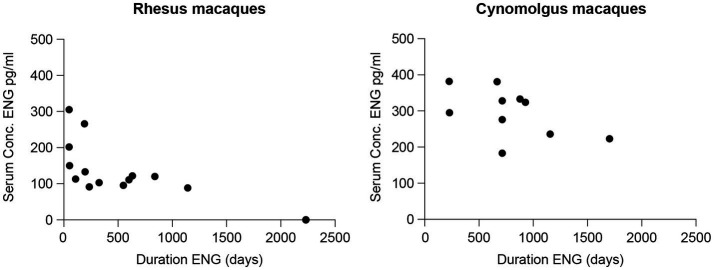
Sera concentrations (pg./mL) of etonogestrel (ENG) in rhesus (left) and cynomolgus macaques (right) with one-third ENG implant in relation to the duration of ENG implant measured in days starting from the day of insertion.

Animal characteristics measured at the time of blood sampling are summarized in Table 3. Individual results are presented in Supplementary Table S1. Figure 2 presents the sera concentrations of the individual animals in relation to the duration of their ENG implant. All animals received a dose of one-third ENG implant. In this study, the maximum duration of ENG with a serum concentration above detection level was 1,142 days (3.1 years) and 1,704 days (4.7 years) for rhesus and cynomolgus macaques, respectively.

**Table 3:** Summary of animal characteristics carrying ENG including age (years), body weight (kilograms), in-treatment duration (days), and ENG serum concentration (pg./mL) measured at the time of blood sampling.

Species (n = number of females)	Age (years) Median (min-max)	Bodyweight (kg) Median (min-max)	Duration (days) Median (min-max)	Serum concentration (pg./mL) Median (min-max)
Rhesus (*n* = 16)	13.5 (6–24)	8.6 (5.7–13.2)	438 (49–2,182)	112 (0–305)
Cynomolgus (*n* = 10)	13.5 (11–22)	6.5 (5.3–9.2)	714 (226–1704)	310 (183–382)

A wide interindividual variability is suspected as three cynomolgus macaques, all carrying ENG for 714 days (1.9 years), showed a serum concentration ranging between 183 and 328 pg./mL. By comparing with a small intraindividual variability, one rhesus macaque was sampled twice, at in-treatment days 633 and 839 (1.7 and 2.3 years) measuring 122 and 120 pg./mL, respectively, a decrease in concentration of 1.6%.

ENG serum concentrations were significantly lower for rhesus macaques compared to cynomolgus macaques (two-sample Mann–Whitney test, *p* < 0.001). In addition, the ENG serum concentration and the duration of ENG implant were significantly correlated in rhesus (Spearman’s *r* = −0.83, *p* < 0.001) compared to a non-significant correlation in the cynomolgus macaques (Spearman’s *r* = −0.55, *p* = 0.100).

In rhesus macaques, the ENG serum concentration was significantly correlated with body weight (kg) (Spearman’s rank *r* = −0.52, *p* = 0.039), but this correlation was not found in cynomolgus macaques (Spearman’s rank *r* = −0.24, *p* = 0.507). Furthermore, a significantly lower body weight was observed for cynomolgus macaques compared to rhesus macaques (two-sample Mann–Whitney test *p* = 0.036).

### 3.7 Hematology and serum chemistry evaluation

Twenty-four rhesus macaques were eligible for inclusion to evaluate possible effects on serum concentrations. There were not sufficient data available for statistical analysis regarding cynomolgus macaques. The mean age for the rhesus macaques at baseline was 8.5 years (range 2.5–22.2), and all animals received a dose of one-third ENG implant. The blood parameters that were used to compare baseline vs. in-treatment timepoint ranges are shown in Table 4. Although serum concentrations of alkaline phosphatase were significantly decreased at >2.5 years post-ENG, they were within the physiological range of the BPRC reference intervals (31). Gamma-glutamyl transferase showed a decreased serum concentration at all timepoints. Creatinine showed a significant increase at all timepoints compared to baseline, all within reference intervals. For hemoglobin, we observed a significant increase starting from 1.5 years post-ENG.

**Table 4:** Blood serum parameters for female rhesus macaques comparing baseline vs. in-treatment period with one-third ENG, 6 months to 1.5 years (>0.5), 1.5–2.5 years (>1.5), and 2.5–3.5 years (>2.5).

Parameter reference intervals	Baseline vs. period	Count	Median (mean) baseline	Median (mean) ENG	Delta	Pw
Albumin (g/L)	>0.5	24	42.0 (41.3)	41.4 (40.8)	−0.56	0.1208
38.66–42.58 (g/L)	>1.5	21	41.9 (41.0)	41.5 (41.1)	0.07	0.6766
	>2.5	15	41.9 (41.0)	40.7 (40.3)	−0.70	0.8040
Alkaline phosphatase (U/L)	>0.5	24	209.9 (308.3)	200.7 (251.7)	−56.68	0.0646
121.5–202.4 (U/L)	>1.5	21	205.2 (296.4)	207.9 (209.2)	−87.22	0.0958
	>2.5	15	**205.2 (318.8)**	**180.2 (173.8)**	**−145.01**	**0.0413**
Alanine transaminase (U/L)	>0.5	24	32.3 (34.2)	29.4 (31.7)	−2.57	0.7048
21.50–40.10 (U/L)	>1.5	21	33.1 (34.3)	29.5 (31.4)	−2.88	0.5315
	>2.5	15	33.1 (33.8)	35.7 (39.3)	5.51	0.2769
Aspartate aminotransferase (U/L)	>0.5	24	34.1 (34.1)	29.5 (31.1)	−3.05	0.2531
23.53–35.30 (U/L)	>1.5	21	33.6 (34.2)	31.5 (30.9)	−3.32	0.4654
	>2.5	15	33.6 (35.5)	37 (48.9)	13.46	0.4953
Total bilirubin (μmol/L)	>0.5	21	1.3 (1.7)	1.2 (1.3)	−0.38	0.1643
0.50–1.50 (μmol/L)	>1.5	19	1.3 (1.7)	1.2 (1.5)	−0.18	0.2759
	>2.5	13	1.8 (2.1)	1.3 (1.9)	−0.24	0.4844
Cholesterol (mmol/L)	>0.5	24	3.5 (3.5)	3.6 (3.6)	0.01	0.9317
3.02–3.81 (μmol/L)	>1.5	21	3.5 (3.5)	3.7 (3.7)	0.16	0.1730
	>2.5	15	3.7 (3.6)	3.9 (3.5)	−0.07	0.9780
Creatinine (μmol/L)	>0.5	24	**65.6 (65.0)**	**68.2 (69.3)**	**4.26**	**0.0146**
67.2–85.20 (μmol/L)	>1.5	21	**65.0 (64.6)**	**79.4 (75.9)**	**11.30**	**0.0025**
	>2.5	15	**58.3 (62.7)**	**77.3 (77.2)**	**14.51**	**0.0103**
Gamma-glutamyl transferase (U/L)	>0.5	24	**54.6 (60.2)**	**52.4 (51.7)**	**−8.57**	**0.0005**
56.10–90.80 (U/L)	>1.5	21	**52.8 (58.9)**	**46.6 (47.9)**	**−10.96**	**0.0001**
	>2.5	15	**48.4 (55.2)**	**44.2 (45.7)**	**−9.44**	**0.0084**
Hemoglobin (mmol/L)	>0.5	24	8.1 (8.0)	8 (8.0)	−0.03	0.9611
7.70–8.40 (mmol/L)	>1.5	21	**8.0 (8.0)**	**8.2 (8.3)**	**0.23**	**0.0140**
	>2.5	14	**8.0 (8.0)**	**8.4 (8.4)**	**0.34**	**0.0079**
Potassium (mmol/L)	>0.5	24	3.5 (3.5)	3.7 (3.6)	0.13	0.0813
3.36–3.76 (mmol/L)	>1.5	21	3.5 (3.5)	3.6 (3.5)	0.04	0.4319
	>2.5	15	3.5 (3.5)	3.6 (3.7)	0.15	0.2680
Sodium (mmol/L)	>0.5	24	145.8 (145.4)	146.2 (146.0)	0.53	0.5485
143.9–147.4 (mmol/L)	>1.5	20	145.3 (145.3)	146.4 (146.0)	0.70	0.3409
	>2.5	15	145.9 (145.2)	146.8 (145.2)	−0.04	0.8904
Total protein (g/L)	>0.5	24	64.2 (64.1)	65.6 (64.3)	0.17	0.7210
60.90–66.20 (g/L)	>1.5	21	64.2 (63.8)	65.6 (65.5)	1.72	0.1137
	>2.5	15	64.7 (64.6)	65.5 (64.7)	0.10	0.8040
Urea (mmol/L)	>0.5	24	6.4 (6.7)	6.9 (6.8)	0.18	0.8415
5.53–7.34 (mmol/L)	>1.5	21	6.1 (6.5)	6.5 (7.1)	0.65	0.1262
	>2.5	15	5.9 (6.4)	6.5 (6.7)	0.28	0.7197

The median and mean values of the parameters are shown prior to ENG insertion (baseline) and in-treatment (ENG). Delta shows the change in mean blood concentration in-treatment compared to the baseline levels. The non-parametric significance (Wilcoxon signed-rank, Pw) levels of these changes are shown as well. Reference intervals (31) are given each parameter. Significant changes are shown in boldface.

### 3.8 Ultrasound

**Figure 3:**
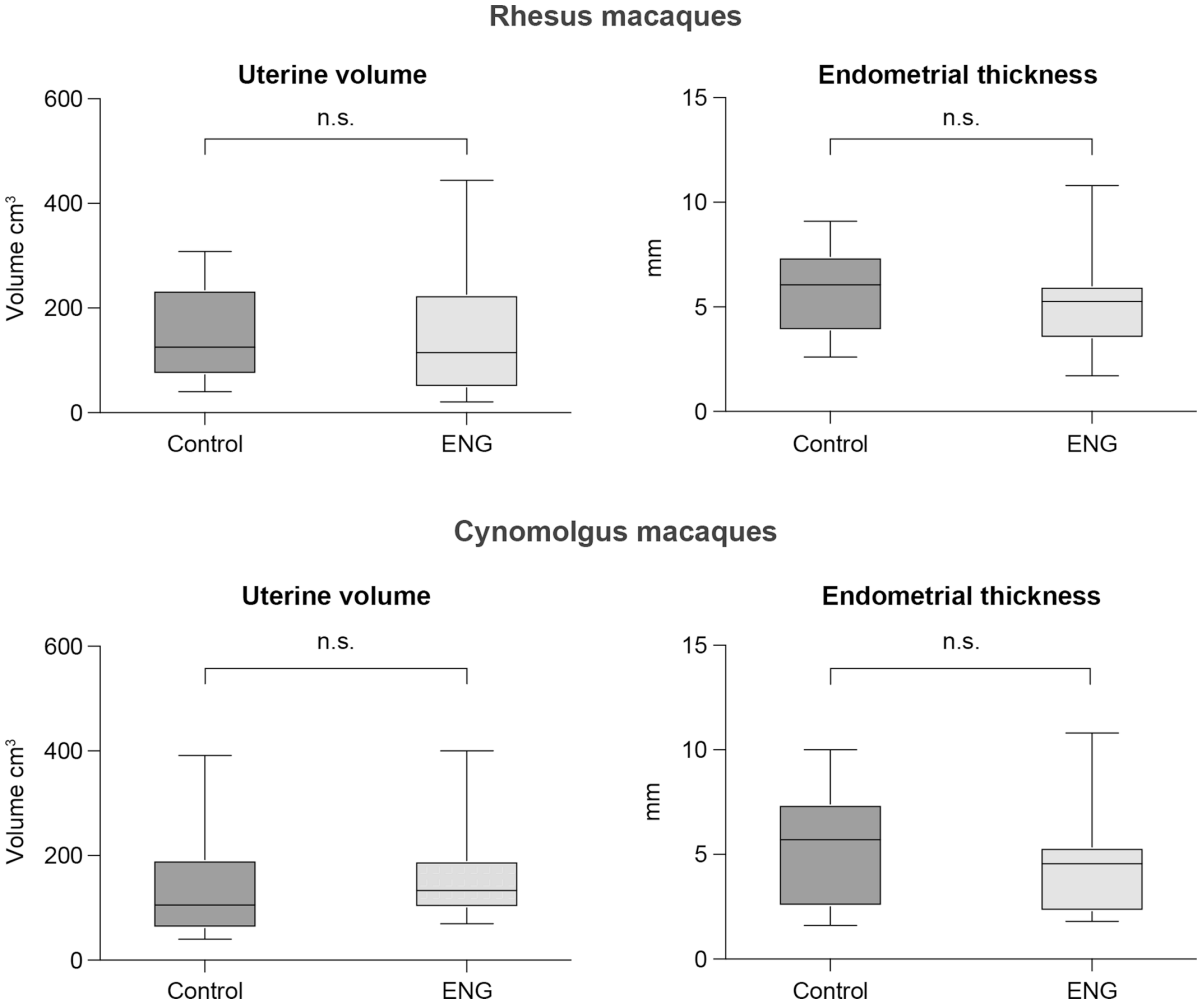
Boxplots showing median, 25 and 75 percentiles, and the whiskers represent minimum and maximum value of uterine volume and endometrial thickness of both female rhesus and cynomolgus macaques. Each plot shows the data from the control group (non-ENG) and the in-treatment ENG females. For each in-treatment animal, a representative control animal is shown.

Data for both species are summarized in Figure 3, and individual animal data are shown in Supplementary Table S2. For the rhesus macaques, the median in-treatment uterine volume was 115 cm^3^ (range 21–444 cm^3^) compared to 125 cm^3^ (range 40–308 cm^3^) for the control animals (Mann–Whitney test, unpaired, two-tailed, *p* = 0.62). The median in-treatment endometrial thickness was 5.3 mm (range 1.7–10.8 mm) compared to 6.1 mm (range 2.6–9.10 mm) for the controls (Mann–Whitney test, unpaired, two-tailed, *p* = 0.32).

For the cynomolgus macaques, the median in-treatment uterine volume was 106 cm^3^ (range 40–391 cm^3^) compared to 133 cm^3^ (range 70–400 cm^3^) for the controls (Mann–Whitney test, unpaired, two-tailed, *p* = 0.24). The median in-treatment endometrial thickness was 4.6 mm (range 1.8–11 mm) compared to 5.7 mm (range 1.6–10) for the controls (Mann–Whitney test, unpaired, two-tailed, *p* = 0.44).

## 4 Discussion

The present study shows that ENG implantable contraception is a reliable, effective, and reversible long-term population management tool in rhesus and cynomolgus macaques. Our data showed that both one-fourth and one-third ENG implants exceeded 99% effectiveness. Typical use of this implant in humans achieves a contraceptive protection exceeding 99%, which is similar to our observations in macaques. Furthermore, comparable to humans, in our study, no adverse reaction to the implant was observed.

Valerio et al. described that overall, 15% of all pregnancies result in an abortion, and 35% of the abortions occurred in the first 2 months of pregnancy in macaques (32). In our study, no abortions were observed in the in-treatment animals. However, due to the physically small size of the aborted embryo or fetus, an abortion during this early stage could have been missed during the daily observations, which might have impacted the efficacy rate of ENG. In addition, embryonic and fetal resorption were possibly unnoticed as pregnancy was only checked by parturition and once a year during routine physical health examinations.

A common challenge for all NHPs is the risk of losing the implant due to active or accidental removal by the individual animal or its social companions, for example, during grooming. Our retrospective study involved one animal in which a potential implant loss was diagnosed.

In humans, to avoid the potential of early pregnancies, ENG implants are advised to be inserted between the first and fifth day of the menstrual cycle (33). In our study, the exact stage of the menstrual cycle was not determined. ENG insertions were usually combined with the annual health examinations, to reduce the discomfort of an additional sedation, and therefore, the ENG treatments started at any possible timepoint within the ovarian cycle. The downside of this approach is that the contraceptive effectiveness is not immediately fully in place but is delayed by approximately 7 days. In our study, one rhesus macaque conceived at approximately in-treatment day 16, and therefore, it can be argued that the ENG concentration was not yet sufficient at the time of conception.

The 3-year contraceptive effectiveness in our study was similar to that found in human studies. Since in women, several studies have demonstrated the prolonged effectiveness of ENG implants for up to 5 years, we also explored the prolonged effectiveness in macaques (9, 34–36). Our results showed an effective prolonged in-treatment range of 1,134–1805 days (3.1–5.0 years) in 14 rhesus macaques and an in-treatment range of 1,154–1,441 days (3.2–4.0 years) in eight cynomolgus macaques. In total, nine macaques had one-fourth and 13 macaques had one-third ENG, suggesting that both dosages have a prolonged effectiveness. As for the one-third dose, the prolonged effectiveness is supported by the serum ENG concentrations observed in both rhesus and cynomolgus macaques. Assuming that the established human threshold concentration of 90 pg./mL to prevent ovulation is also applicable to macaques, three out of six animals maintained ENG serum concentrations above 90 pg./mL when the 3-year in-treatment period was exceeded (Supplementary Table S1). However, the three other rhesus females showed no detectable ENG levels at in-treatment day 2,231 (6.1 years). Although our study comprised a small cohort, the results suggest that, in line with human literature, ENG can be an effective contraception beyond 3 years and up to 5 years. In addition, caution must be applied as the one-fourth dose was not evaluated by serum ENG concentrations.

Existing human data about the effect on body weight from ENG are limited and inconclusive (37). Whereas body weight development is multifactorial, factors such as age, social rank, and diet are known to have considerable effect on body weight in macaques (38, 39). Evaluation of the in-treatment effect of ENG on body weight was, therefore, beyond the scope of this study.

In women, the ENG dose is one complete implant (68 mg ENG) inserted subcutaneously, and this results in a dose of 0.97 mg/kg presuming a 70 kg standard human body weight. Although at the BPRC, only one-fourth or one-third part of the implant was inserted in each macaque, this is still a higher dose compared to the human dose. By using the one-third implant in macaques weighing 5.3–13.2 kg, the dose was 4.5–1.7 mg/kg, establishing a serum concentration > 90 pg./mL. In our study, 108 animals received one-fourth part and 137 animals one-third part implanon, no side effects of overdosing were observed. In addition, no differences were observed between doses (one-fourth—one-third part) and effectiveness.

To quantify ENG sera concentration released from one-third part of an ENG implant, endocrine monitoring using hormone assays was applied. This cross-sectional analysis showed a significantly lower ENG serum concentration and higher body weight for rhesus macaques compared to cynomolgus macaques. The relationship between higher body weights and ENG concentrations has been investigated in humans. These study outcomes suggested that BMI had no or limited influence on the contraceptive effectiveness of ENG (12, 14). In addition, the ENG concentration in overweight and obese women remained at approximately 90 pg./mL regardless of BMI (12, 13). In contrast, Lazorwitz et al. described a decrease in ENG concentration in relation to increased BMI, and Mornar et al. observed a lower concentration in obese women compared to their normal counterparts (12, 40). However, Morrell et al. observed a slight increase in ENG concentration in overweight women and none in obese women (13). Although two rhesus and nine cynomolgus macaques were overweight, these animals had also an ENG concentration of at least 90 pg./mg. The body weight between rhesus and cynomolgus macaques differed ranging from 5.7–13.2 kg (median 8.6 kg) and 5.3–9.0 kg (median 6.5 kg), respectively. Both species received a similar dose (one-third part) of ENG resulting in an average higher ENG concentration in the cynomolgus macaques likely due to their lower body weight. Moreover, the difference in concentration between the two species could also be explained by the variability or small sample size.

*In vitro* data have shown that ENG is metabolized in liver microsomes by the cytochrome P450 (CYP450) 3A4 isoenzyme. In addition, Lazorwitz et al. showed that in addition to BMI and duration of the implant, a genetic variant of CYP450 may alter the metabolism of steroid hormones, and thus the serum etonogestrel concentrations (41). The cynomolgus macaque CYP450 3A homolog and the human CYP450 3A are known to have similar substrate selectivity (42). In addition, rhesus macaques CYP450 exhibit 93% homology of the amino sequence similarity with human CYP450 3A4 (43). Inter-animal variations in cynomolgus macaques for CYP450 were observed, generally 3–4-fold for most CYP450s, including CYP3A4, and this degree of variation was much less than in humans (44). Although variation in CYP450 was not evaluated in the current study, in accordance with Lazorwitz et al., we observed an inter-animal variability in ENG concentration between the animals carrying ENG for a similar duration (40, 41). Furthermore, the CYP450 3A homolog in rhesus and cynomolgus macaques are 100% identical at the amino acid level and 99% identical at mRNA sequence and, therefore, an unlikely explanation for the differences in ENG serum concentration between both species (43, 45).

Despite our relatively small study population regarding reversibility, all 14 cynomolgus macaques had parturition after implant removal and three rhesus macaques even without the removal of an expired implant. In the cynomolgus macaques, the earliest conception occurred approximately 18 days after the removal of the implant. These results agree with the findings in humans, in which pregnancies were reported as soon as 7–31 days after ENG removal (34). Therefore, we conclude that ENG is also reversible in macaques.

In humans, some hematological and biochemical parameters are described to change with the use of ENG, but they were described as non-significant enough to cause clinical sequelae (46–49). In rhesus macaques, the biochemical parameters, such as alkaline phosphatase and gamma-glutamyl transferase, showed a significantly decreased serum concentration and creatinine showed an increased concentration compared to baseline, but all parameters stayed within published reference intervals (31). In humans, during the luteal phase, the glomerular filtration and creatinine clearance are increased (50). In macaques, however, no literature is available regarding this phenomenon. The increased creatinine levels, though within reference intervals, combined with unchanged urea levels make early renal failure unlikely. Unfortunately, there were not sufficient data available for statistical analysis in cynomolgus macaques.

In line with Dilbaz et al. and Guazeelli et al., the hemoglobin concentration in female rhesus macaques was increased compared to baseline (47, 48). It was suggested that in humans, this was due to reduced or absent bleeding patterns in their population. In group-housed macaques, it is challenging to score menses, and therefore, bleeding pattern observations were not performed in the current study. However, the absence or reduction in bleeding pattern is likely to occur as well in rhesus macaques with ENG, and additional blood loss during (yearly) parturition is also prevented. Possibly, both occurrences have added to the increased hemoglobin concentration.

As previously mentioned, clinical signs of the ovarian cycle of macaques, such as menses and perineal skin tumescence, were not included in our study. These observations cannot be generalized as rhesus macaques, which are described to have seasonality with anovulatory cycles in the non-breeding season, still exhibit menstruation (15, 51, 52). In addition, an early sign of conception in macaques is implantation bleeding, which can be mistaken for normal menses (32). In addition, perineal sex skin color changes and swelling fluctuations do not occur in all animals and can therefore not be used as a parameter to monitor ovarian cyclicity in macaques (15).

In Barbary macaques, ENG can result in increased aggression, more self-scratching, and more time self-grooming, demonstrating anxiety (53). Assessing the contraceptive efficacy of ENG was not included in their study. In our colony, animals were observed twice daily by a caretaker for (ab)normal (sexual) behavior. Although not studied in full detail, no abnormal behavior related to ENG was noted in our study. In addition, ENG treatment of some individuals did not disrupt the social dynamics in our group-housed macaques.

Although it is recommended that pregnancy is excluded prior to implant insertion, there is no evidence that ENG has effects on established pregnancies (17, 37). In addition, based on human data, ENG may be used during lactation (10). In our study, three in-treatment parturitions resulted in two viable neonates. However, teratogenic studies showed no evidence of fetal harm when exposed to high-dose ENG exposure in rats and rabbits.

At the BPRC, ENG has been used as a contraceptive in multiple female macaques. In addition to contraception, ENG was also used as a clinical treatment for hormonally responsive diseases such as endometriosis and menorrhagia in macaques. Endometriosis occurs naturally in NHPs, most commonly, macaques and baboons (54, 55). Symptoms are similar to those described in women (56). In humans, ENG implants have been recommended to relieve abnormal uterine bleeding and pelvic pain associated with endometriosis and leiomyomas (7, 33, 57–62). Currently, it is unknown if these observations can be extrapolated to macaques. Although beyond the scope of the current study, it would be interesting to investigate the feasibility of ENG in macaques with such hormonal-responsive diseases.

Next, to ovulation prevention, a second contraceptive effect of ENG use in humans is a decrease in endometrial thickness (63, 64). Furthermore, a reduction in uterine volume in women carrying ENG has been described (65). In contrast to these studies, the endometrial thickness and the volume of the uterus were not affected by ENG in macaques. The apparent absence of effects could be related to the relatively small number of in-treatment macaques or the variability in the ovarian cycle of the control animals. Although we group-matched the in-treatment animals with control animals as precisely as possible in order of importance, similar parity, age, and, for rhesus macaques, seasonality as well, a longitudinal approach is recommended for future studies in which the animals can serve as their own control (32, 66). Instead of the cross-sectional approach used in our study, longitudinal follow-ups are expected to be more accurate in determining ENG-induced changes in endometrial thickness and uterine volume in individual macaques.

In the present study, we evaluated the use of ENG as long-acting and reversible contraception in a macaque colony consisting of both rhesus and cynomolgus macaques. We conclude that one-fourth—one-third part of ENG implants are effective and safe to use in macaques. In addition, our findings provide some evidence of prolonged effectiveness for up to 5 years. Taken together, these results suggest an important role for the use of ENG as a contraceptive in macaques for population control in zoos, in the wild, and in research colony management.

## Data availability statement

The raw data supporting the conclusions of this article will be made available by the authors, without undue reservation.

## Ethics statement

The animal study was approved by the Institutional Animal Welfare Body (IvD023A). The study was conducted in accordance with the local legislation and institutional requirements.

## Author contributions

AM: Conceptualization, Data curation, Formal analysis, Investigation, Methodology, Validation, Visualization, Writing – original draft, Writing – review & editing. KS: Writing – review & editing, Methodology, Validation. LM: Writing – review & editing, Investigation, Validation. SR: Writing – review & editing, Data curation, Investigation. AL: Writing – review & editing, Data curation, Investigation. ER: Formal analysis, Validation, Writing – review & editing. JL: Writing – review & editing, Supervision. MS: Methodology, Supervision, Validation, Writing – review & editing. JB: Writing – review & editing, Conceptualization, Methodology, Supervision, Writing – original draft.

## Footnotes

^1^https://www.fda.gov/regulatory-information/search-fda-guidance-documents/bioanalytical-method-validation-guidance-industry
