# Supplementary material for: Long-acting reversible contraception with etonogestrel implants in female macaques (Macaca mulatta and Macaca fascicularis)
Source: Front Vet Sci. 2024 Jan 8;10:1319862. doi: 10.3389/fvets.2023.1319862 (PMC10800480; doi:10.3389/fvets.2023.1319862)
Supplement: Supplementary file 1 [file Data_Sheet_1.docx]

Supplementary Material

Long-acting reversible contraception with etonogestrel implants in female macaques (Macaca mulatta and Macaca fascicularis)

Maaskant A.^1,3^*, Scarsi Kimberly .K.^2^, Meijer L.^1^, Roubos S.^1^, Louwerse A.L.^1^, Remarque E.J. ^1^, Langermans J.A.M.^1,3^, Stammes M.A.^1^, Bakker J.^1^

*** Correspondence:** Annemiek Maaskant; maaskant@bprc.nl

# Supplementary Tables

Supplementary Table 1. ID and individual animal characteristics for both rhesus (RM) and cynomolgus macaques (CM), insertion date (day/month/year) of etonogestrel implant, collection date of blood sample used for analysis, duration of in-treatment period at sample collection, serum concentration (pg/mL) levels and when below detection BLQ, weight-for-height index (WHI) and bodyweight.

| **ID** | **Group** | **Birth Date** | **Insert ENG** | **Blood Sample** | **Duration**  **ENG** | **ENG**  **pg/mL** | **Weight**  **(kg)** | **WHI** |
| --- | --- | --- | --- | --- | --- | --- | --- | --- |
| RM1 | CONTROL | 20/06/2006 | - | 10/11/2021 | - | BLQ | 8 | 79 |
| RM2 | CONTROL | 03/05/2016 | - | 10/11/2021 | - | BLQ | 13.5 | 64 |
| RM3 | ENG | 12/05/1998 | 25/03/2021 | 15/11/2021 | 235 | 92 | 13.1 | 92 |
| RM4 | ENG | 02/07/2007 | 18/10/2019 | 19/04/2021 | 549 | 96 | 9.9 | 62 |
| RM5 | ENG | 20/07/2006 | 16/10/2015 | 24/11/2021 | 2231 | BLQ | 13.2 | 79 |
| RM6 | ENG | 13/06/2006 | 09/06/2021 | 28/07/2021 | 49 | 202 | 7 | 46 |
| RM7 | ENG | 20/05/2005 | 28/05/2021 | 20/07/2021 | 53 | 150 | 9.6 | 50 |
| RM8 | ENG | 08/08/2003 | 07/10/2020 | 16/04/2021 | 191 | 266 | 8.4 | 58 |
| RM9 | ENG | 16/06/2002 | 25/03/2021 | 12/07/2021 | 109 | 113 | 11.2 | 64 |
| RM10 | ENG | 09/06/2015 | 01/08/2019 | 24/03/2021 | 601 | 111 | 8.8 | 56 |
| RM11 | ENG | 30/04/2015 | 25/10/2019 | 10/02/2022 | 839 | 120 | 5.7 | 43 |
| RM11 | ENG | 30/04/2015 | 25/10/2019 | 19/07/2021 | 633 | 122 | 5.7 | 43 |
| RM12 | ENG | 25/05/2010 | 16/10/2015 | 24/11/2021 | 2231 | BLQ | 10.3 | 60 |
| RM13 | ENG | 03/05/2010 | 15/10/2018 | 30/11/2021 | 1142 | 88,4 | 8.2 | 60 |
| RM14 | ENG | 14/06/2009 | 28/05/2020 | 19/04/2021 | 326 | 103 | 7.5 | 47 |
| RM15 | ENG | 06/04/2009 | 10/05/2021 | 22/11/2021 | 196 | 133 | 6.5 | 49 |
| RM16 | ENG | 13/05/2008 | 09/06/2021 | 28/07/2021 | 49 | 305 | 6.1 | 39 |
| RM17 | ENG | 11/04/2008 | 16/10/2015 | 24/11/2021 | 2231 | BLQ | 9.1 | 56 |
| CM1 | CONTROL | 08/06/2005 | - | 05/07/2021 | - | BLQ | 5.6 | 67 |
| CM2 | CONTROL | 25/05/2014 | - | 14/07/2021 | - | BLQ | 4 | 43 |
| CM3 | ENG | 02/04/2008 | 13/10/2020 | 27/05/2021 | 226 | 382 | 6.8 | 66 |
| CM4 | ENG | 11/02/2007 | 03/01/2019 | 31/05/2021 | 879 | 333 | 5.5 | 64 |
| CM5 | ENG | 03/04/2007 | 17/06/2019 | 31/05/2021 | 714 | 276 | 6.3 | 76 |
| CM6 | ENG | 17/08/2007 | 14/10/2020 | 31/05/2021 | 229 | 295 | 8 | 76 |
| CM7 | ENG | 21/01/2008 | 17/06/2019 | 31/05/2021 | 714 | 328 | 8.4 | 74 |
| CM8 | ENG | 31/03/2008 | 17/06/2019 | 31/05/2021 | 714 | 183 | 9.2 | 86 |
| CM9 | ENG | 09/08/1999 | 01/05/2018 | 30/06/2021 | 1156 | 236 | 6.3 | 72 |
| CM10 | ENG | 01/02/2004 | 02/09/2019 | 30/06/2021 | 667 | 381 | 6.0 | 67 |
| CM11 | ENG | 06/07/2008 | 21/12/2018 | 07/07/2021 | 929 | 324 | 7.2 | 72 |
| CM12 | ENG | 18/02/2010 | 15/08/2016 | 15/04/2021 | 1704 | 223 | 5.3 |  |

Supplementary Table 2. Ultrasound data for the individual rhesus macaques (RM) and cynomolgus macaques (CM) carrying ENG and the matched control group (CONTROL). Parity is shown in number of parturition at time of ultrasound procedure, age (years), volume of the uterus (cm^3^) and the endometrial thickness (mm). For RMs the breeding season was included in the matching process, the (1) breeding season was set from October - March and (2) non-breeding season from April – September .

| **ID** | **Group** | **Parity** | **Age** | **Volume (cm^3^)** | **Endometrium (mm)** | **Season** |
| --- | --- | --- | --- | --- | --- | --- |
| RM3 | ENG | 6 | 24 | 85.1 | 1.7 | 2 |
| RM5 | ENG | 2 | 15 | 130.1 | 5.5 | 2 |
| RM8 | ENG | 9 | 18 | 303.6 | 10.8 | 1 |
| RM9 | ENG | 7 | 19 | 377.9 | 3.2 | 1 |
| RM11 | ENG | 0 | 6 | 20.5 | 3.4 | 1 |
| RM12 | ENG | 2 | 12 | 183.1 | 5.2 | 2 |
| RM13 | ENG | 1 | 12 | 50.9 | 5.4 | 2 |
| RM14 | ENG | 4 | 12 | 25.4 | 3.5 | 1 |
| RM15 | ENG | 4 | 13 | 99.5 | 6 | 2 |
| RM16 | ENG | 8 | 13 | 38 | 4.5 | 1 |
| RM17 | ENG | 2 | 14 | 200.4 | 5.3 | 2 |
| RM18 | ENG | 3 | 13 | 54.4 | 5 | 2 |
| RM19 | ENG | 5 | 15 | 444.3 | 6.6 | 1 |
| RM20 | ENG | 6 | 25 | 175.2 | 6 | 1 |
| RM21 | CONTROL | 0 | 6 | 40.1 | 3.7 | 1 |
| RM22 | CONTROL | 1 | 10 | 308.1 | 5.7 | 2 |
| RM23 | CONTROL | 2 | 11 | 106.2 | 4.9 | 2 |
| RM24 | CONTROL | 2 | 10 | 76.5 | 3.2 | 2 |
| RM25 | CONTROL | 2 | 8 | 55 | 6.4 | 2 |
| RM26 | CONTROL | 3 | 12 | 243.6 | 8.7 | 2 |
| RM27 | CONTROL | 4 | 14 | 102 | 3.9 | 2 |
| RM28 | CONTROL | 4 | 12 | 144 | 9.1 | 1 |
| RM29 | CONTROL | 5 | 11 | 75.6 | 6.9 | 1 |
| RM30 | CONTROL | 6 | 16 | 234.9 | 7.4 | 1 |
| RM31 | CONTROL | 8 | 14 | 151.7 | 5 | 1 |
| RM32 | CONTROL | 9 | 17 | 64.9 | 6.5 | 1 |
| RM33 | CONTROL | 6 | 16 | 234.9 | 7.4 | 1 |
| RM34 | CONTROL | 6 | 15 | 214.9 | 2.6 | 2 |
| CM3 | ENG | 4 | 13 | 105.5 | 6.7 |  |
| CM4 | ENG | 1 | 14 | 51.2 | 1.8 |  |
| CM5 | ENG | 2 | 14 | 60.7 | 4.7 |  |
| CM6 | ENG | 1 | 14 | 307.8 | 4.4 |  |
| CM7 | ENG | 1 | 13 | 108.2 | 1.9 |  |
| CM8 | ENG | 1 | 13 | 192.4 | 10.8 |  |
| CM9 | ENG | 1 | 22 | 40.2 | 4.9 |  |
| CM10 | ENG | 7 | 17 | 60.7 | 2.4 |  |
| CM11 | ENG | 4 | 13 | 169.4 | 4.9 |  |
| CM12 | ENG | 2 | 11 | 72.2 | 3 |  |
| CM13 | ENG | 0 | 27 | 391.2 | - |  |
| CM14 | CONTROL | 4 | 13 | 224 | 6.4 |  |
| CM15 | CONTROL | 1 | 14 | 173.4 | 1.6 |  |
| CM16 | CONTROL | 2 | 12 | 171.5 | 10 |  |
| CM17 | CONTROL | 1 | 8 | 69.5 | 2.4 |  |
| CM18 | CONTROL | 1 | 10 | 119.8 | 2.5 |  |
| CM19 | CONTROL | 1 | 12 | 400.1 | 9.9 |  |
| CM20 | CONTROL | 1 | 14 | 190.1 | 2.9 |  |
| CM21 | CONTROL | 7 | 16 | 100.3 | 6.4 |  |
| CM22 | CONTROL | 4 | 12 | 95 | 7.4 |  |
| CM23 | CONTROL | 2 | 11 | 123.9 | 5.6 |  |
| CM24 | CONTROL | 0 | 18 | 133 | 5.7 |  |

**
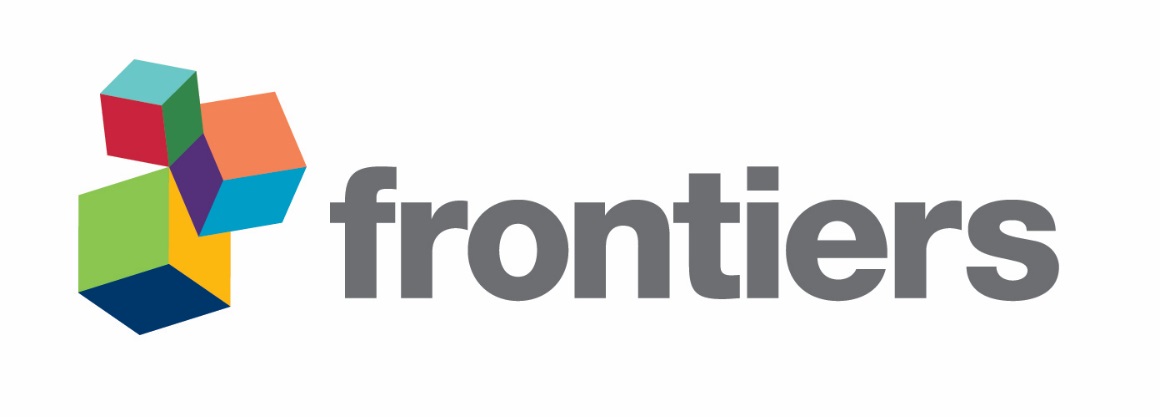
**
